# Locally advanced NSCLC: a plea for sparing the ipsilateral normal lung—prospective, clinical trial with DART-bid (dose-differentiated accelerated radiation therapy, 1.8 Gy twice daily) by VMAT

**DOI:** 10.1186/s13014-022-02083-6

**Published:** 2022-07-07

**Authors:** Karl Wurstbauer, Margit Kazil, Marco Meinschad, Raoul Pinter, Catharina De Vries, Patrick Clemens, Christof Kreuter, Tamara Hernler, Wolfgang Hitzl, Peter Cerkl, Thomas Künzler, Alexander De Vries

**Affiliations:** 1 Department for Radiation Oncology, Academic Teaching Hospital, Carinagasse 47, 6800 Feldkirch, Austria; 2 Academic Teaching Hospital, Institute of Medical Physics, Feldkirch, Austria; 3 Department for Pneumology, Academic Teaching Hospital, Hohenems, Austria; 4 Team Biostatistics and Publication of Clincial Studies, FM&TT, Paracelsus Medical University, Salzburg, Austria

**Keywords:** Lung cancer, Locally advanced NSCLC, VMAT, DART-bid, Radiation dose intensification, QUANTEC, Margins, Planning

## Abstract

**Background:**

In radiation treatment of locally advanced non-small cell lung cancer (LA-NSCLC), ‘margins’ from internal target volumes to planning target volumes in the range of 12 to 23 mm are reported, and avoiding exposure of the contralateral lung is common practice. We investigated prospectively an approach with tight margins (7 mm) and maximal sparing of the ipsilateral normal lung. Mature results for the first endpoint (pneumonitis) and further toxicities are reported.

**Methods:**

Primary tumors were treated by VMAT with 73.8–90.0 Gy in positive correlation to tumor volumes, nodes with 61.2 Gy, a restricted volume of nodes electively with 45 Gy. Fractional doses of 1.8 Gy bid, interval 8 h. Before radiotherapy, two cycles platin-based chemotherapy were given. 12 patients finished maintenance therapy with Durvalumab. Median follow up time for all patients is 19.4 months, for patients alive 27.0 months (3.4–66.5 months).

**Results:**

100 consecutive, unselected patients with LA-NSCLC in stages II through IVA were enrolled (UICC/AJCC, 8th edition). No acute grade 4/5 toxicity occurred. Pneumonitis grade 2 and 3 was observed in 12% and 2% of patients, respectively; lowering the risk of pneumonitis grade ≥ 2 in comparison to the largest study in the literature investigating pneumonitis in LA-NSCLC, is significant (p < 0.0006). Acute esophageal toxicity grade 1, 2 and 3 occurred in 12%, 57% and 3% of patients, respectively. Two patients showed late bronchial stricture/atelectasis grade 2. In two patients with lethal pulmonary haemorrhages a treatment correlation cannot be excluded. Median overall survival for all stage III patients, and for those with ‘RTOG 0617 inclusion criteria’ is 46.6 and 50.0 months, respectively.

**Conclusions:**

Overall toxicity is low. In comparison to results in the literature, maximal sparing the ipsilateral normal lung lowers the risk for pneumonitis significantly.

***Trial registration*:**

Ethics committee of Vorarlberg, Austria; EK-0.04-105, Registered 04/09/2017—Retrospectively registered. http://www.ethikkommission-vorarlberg.at

## Introduction

About 85% of lung cancers are histologically non-small cell cancers (NSCLC); and about one third of them present in locally advanced stages initially (LA-NSCLC).

External radiotherapy is a cornerstone in treating LA-NSCLC. Reported margins from ITV to PTV range between 18 and 23 mm in 3D-conformal series [1–3], and between 12 and 15 mm in IMRT/VMAT series [4–6]. Furthermore, in common practice within the radiation planning and delivering process, the contralateral lung usually is spared from exposure. This is reflected by descriptions as ‘dose to the contralateral lung was kept as low as possible’, or ‘beams' distributions were generally partial to one side of the lung with tumors, sparing contralateral lung as far as possible’ [7, 8].

In contrast, an approach was published emphasizing sparing of the ipsilateral normal lung. It was achieved by relatively tight margins (7 mm from ITV to PTV) and appropriate dose distributions, taking into account also exposure of non-lung tissues as spine and anterior mediastinum, and also the contralateral lung (’target splitting’, an advanced 3D- conformal mode) [9]. Differentiated doses up to 90 Gy were applied in an accelerated mode, combined with chemotherapy sequentially (DART-bid by target splitting) [10]. Toxicity, in particular pulmonary toxicity was moderate.

In order to investigate this approach using the technique of volumetric arc therapy (VMAT), a prospective clinical trial in January 2015 was started. Primary planning objective was maximal sparing of the ipsilateral healthy lung tissue; primary endpoint is acute pulmonary toxicity (pneumonitis), secondary endpoints are further toxicity, loco-regional tumor control and survival. Principal treatment parameters as differentiated target doses, fractional doses and sequential combination with chemotherapy were adopted unchanged from the target splitting protocol; whereas the technique of target splitting was replaced by VMAT. The largest study evaluating radiation pneumonitis in LA-NSCLC at that time was chosen as comparative cohort: a meta-analysis of > 800 patients treated with 60 Gy [11]. The hypothesis is to demonstrate that the risk for pneumonitis grade ≥ 2 is significantly lower as compared to the comparative patients, albeit the application of higher target doses.

In this article, the primary end point of the trial (pneumonitis) and further toxicity of 100 consecutive patients are reported, and issues regarding maximal ipsilateral lung sparing in high dose radiotherapy are discussed. In-depth results for loco-regional tumor control and survival will be given in a future report.

## Methods

### Trial design

Eligible patients had non-resected, histologically/cytologically proven NSCLC in stages II (medically inoperable), III A-C and IVA (if stage IVA was determined by separate tumor nodule(s) in a contralateral lobe) and a Karnofsky Index of ≥ 50. As in the RTOG 0617 trial, patients with an undetectable NSCLC primary, but with pathologically proven NSCLC nodes were also eligible; and also patients after treatments of lung cancer in the past, being affected by a second lung cancer—not patients with recurrences of prior tumors. Patients were staged according to the 8th edition of UICC/AJCC classification.

In case of no medical contraindications, 2 cycles of chemotherapy with platin-based doublets were given prior to radiotherapy. Choice of single agents was left to the discretion of referring departments.

With a target interval of < 8 days, radiotherapy was given twice daily with fractional doses of 1.8 Gy (ICRU), with an interval of 8 h, 5 days/week. Overall doses to primary tumors were aligned along increasing tumor volumes within 4 groups in the range of 73.8–90.0 Gy (Table 2; of note, primary tumor diameters are specified as mean of three perpendicular diameters, not as maximal diameters). Dose to macroscopically involved nodes was 61.2 Gy; to elective nodes 45.0 Gy. PTV of elective irradiation was kept small, i.e. limited to nodal sites approximately 6 cm cranial of macroscopic involvement, bilateral for left-sided and just ipsilateral for right-sided tumors, with tight margins (5–7 mm).

For patients with PD-L1 (programmed death ligand 1) expression ≥ 1, positive results of the Pacific trial led to the approvement of Durvalumab as maintenance therapy after concurrent and also sequential platin-based chemo-radiation by the European Medicines Agency [12]. Therefore in a major revision of the protocol, maintenance therapy with Durvalumab was introduced for appropriate patients.

In order to assess tolerability as accurately as possible, patients were followed by a physician twice a week during the treatment period. A stopping rule was established as follows: A dose-specified treatment arm (Table 2) would be closed prematurely, if ≥ grade 4 toxicity was scored in 2 patients. In this case the subsequent patients would be treated within the adjacent treatment arm with a lower dose.

### Staging procedures

Staging evaluations included a medical history, physical examination, chest X-ray, 18-fluorodeoxyglucose-positron emission tomography (FDG-PET), bronchoscopy, mostly combined with endoscopic ultrasound with cyto-/histological assessment of mediastinal nodes [13]; and a CT (computed tomography) or MRI (magnetic resonance imaging) of the brain.

### Radiotherapy planning

4D-planning CTs were performed under standardised vacuum immobilisation cradle system, with patients normally breathing. In contouring the lungs as organs at risk, gross tumor volume (GTV) was excluded from lung volume. The proximal bronchial tree was contoured as one structure according to a contouring-atlas [14]. The esophagus was contoured from the level of cricoid cartilage to the gastroesophageal junction. ‘Atlas-conform’ heart contouring was performed [15].

An ITV of the primary tumor and involved nodes was created from gross tumor depictions in the phases of the 4D-planning CT. A margin of 7 mm was added to the ITV to draw the PTV. An extra-margin for a clinical target volume (CTV) was not considered, as a sufficient dose to the rim of microscopic tumor spread would already be applied in high dose radiotherapy. In patients receiving chemotherapy, PTV was delineated at post-chemotherapy scans.

**Fig. 1:**
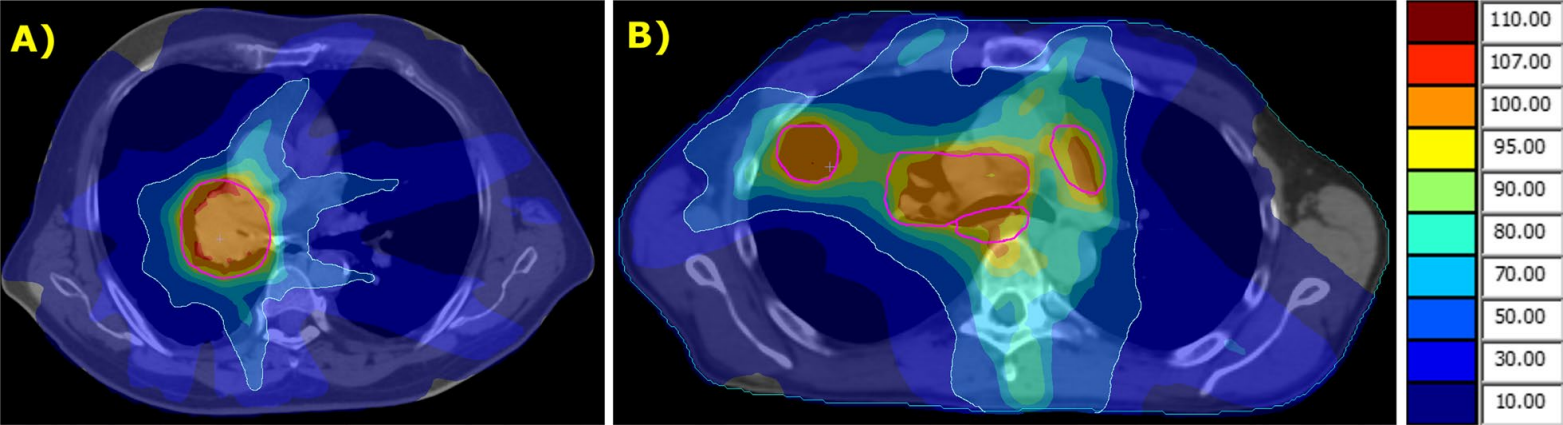
Examples of pulmonary dose distributions (fractional doses). **A**. Centrally located tumor. **B**. Peripheral tumor with hilar and bilateral mediastinal nodes. PTVs are delineated in red; other colors represent percent isodoses—the 50% isodose is marked in light blue

Treatment planning was performed with a Monte Carlo–based planning system (Monaco, Elekta). Minimizing radiation to the ipsilateral lung at the intermediate and high dose level was the primary organ at risk (OAR) sparing objective. The secondary OAR objective was sparing of the esophagus, the tertiary was the contralateral lung. Examples are given in Fig. 1—it’s important to realize that these are slices of the highest exposure; cranial and/or caudal regions of the lung will be less exposed.

### Dose volume specifications

We built up from experiences with DART-bid by target splitting, a technique also aiming to minimize dose to ipsilateral lung [9]. Herein, patients were treated with bilateral pulmonary V20 (volume of lung exposed to ≥ 20 Gy) up to 50%, showing good tolerability, not indicating a pulmonary volumetric threshold (lateral, basal lower lobe tumors excluded; see remarks in ‘discussion’). Therefore, strict pulmonary dose constraints were not given. A dose constraint for spinal cord was set at 45 Gy; and for the esophagus at 80 Gy. In order to not jeopardize the dose coverage of tumors, constraints for the heart and for the proximal bronchial tree were not given; however, an effort to spare these structures was endeavored as far as possible.

Treatments were applied by 6 MV photons. Cone-beam CTs for set-up corrections were performed before every fraction [16].

### Follow-up procedures

Patients were reviewed for assessment of toxicity and tumor control at 2, 6 and 12 weeks after the end of radiotherapy, then every 3 months for the first year, every 4 months during the second and third year, and thereafter every 6 months. At the first and second control chest X-rays, and at all other controls thoracic CTs were performed.

Acute and late toxicities were scored according to the Common Terminology Criteria for Adverse Effects (CTCAE), version 4.03. As pneumonitis as an acute side effect can be present until up to 6 months after therapy, toxicity is considered acute presenting within this period; and is considered as late, if it persisted or developed beyond 6 months after the completion of radiotherapy.

### Statistical analysis

Data were checked for consistency and normality. Overall survival rates were calculated using the Kaplan–Meier method. Fisher’s Exact test was used to compare the risk for acute pneumonitis with grade ≥ 2 in our study with published results. An aposteriori sample size analysis was done to compute the corresponding power for the primary hypothesis.

All statistical tests were done two-sided. All statistical analyses in this report were performed by use of STATISTICA 13 (Hill, T. and Lewicki, P. Statistics: Methods and Applications. StatSoft, Tulsa, OK) and StatXact (2013), Version 10.0.0, Cytel software cooperation (Cambridge, MA, USA).

All time intervals refer to the start of therapy, induction chemotherapy included.

### Ethical statements

The trial was conducted in accordance with the provisions of Declaration of Helsinki (in its current, revised form). It was approved by the institutional review board and by the medical ethics committee of Vorarlberg, Austria, where it was registered under number EK-0.04-105. All patients provided written informed consent.

## Results

100 patients were enrolled between January 2015 and November 2020. These patients correspond to > 90% of all referred LA-NSCLC patients; the majority of not enrolled patients had Pancoast tumors or a Karnofsky Index < 50. Therefore the study population can be considered as an unselected, ‘real life’ cohort. Patient and tumor characteristics are shown in Table 1. Three patients were referred with an undetectable primary NSCLC.

**Table 1:** Patient and tumor characteristics (n = 100)

Age, years, median	68 (43 – 88)
Sex: m/f, n	67 / 33
Weight loss > 5%/3 month, n (%)	29 (29)
Karnofsky Index, n (%)	
100	14 (14)
90	26 (26)
80	37 (37)
70	12 (12)
60	9 (9)
50	2 (2)
COPD as comorbidity, n (%)	
Grade 2	34 (34)
3	17 (17)
4	2 (2)
Stage–grouping (UICC/ AJCC 8th edition), n (%)	
II A	2 (2)
II B	10(10)
III A	27 (27)
III B	43 (43)
III C	14 (14)
IV A*	4 (4)
Histology/cytology, n (%)	
Adenocarcinoma	45 (45)
Squamous cell carcinoma	51 (51)
NSC—n. o. s	4 (4)
Tumor localisation, n (%)	
Central	37 (37)
Peripheral	60 (60)
Primary not detectable	3 (3)
Gross tumor volume, ccm, median (range)	
Group 1 (n = 17)	18 (4 – 47)
Group 2 (n = 52)	45 (12 – 218)
Group 3 (n = 20)	104 (46 – 255)
Group 4 (n = 8)	241 (189 – 387)

*Patients with separate tumor nodule(s) in a contralateral lobe (M1a)

COPD = Chronic obstructive pulmonary disease, UICC = Union for international cancer control, AJCC = American Joint Commission on Cancer; FDG-PET = 18-fluorodeoxyglucose-positron emission tomography; NSC—n.o.s. = Non-small cell—not otherwise specified

Treatment parameters are shown in Table 2. Because of low general conditions—not primarily due to dose-volume causes—three (out of eight patients) in group 4 were treated with 84.6 Gy instead of 90.0 Gy. All other patients received the scheduled overall doses for primary tumors. For the same reason, one patient’s nodes were treated with 57.6 Gy instead of 61.2 Gy. Hence the doses foreseen in the protocol were applied in 96% of the patients. In eight patients, the nodes in close vicinity to primary tumors were included in the PTV of the primary tumors and treated with doses of primary tumors.

**Table 2:** Treatment characteristics

*Total dose (Gy)*	
Primary tumor	
Group 1 (PT-Ø* < 2.5 cm, n = 17)	73.8
Group 2 (2.5 – 4.5 cm, n = 52)	79.2
Group 3 (4.5 – 6.0 cm, n = 20)	84.6
Group 4 (> 6.0 cm, n = 8)	90.0
Nodes	61.2
Nodes electively**	45
*Fractional dose (Gy)*	1.8 bid
Interval	8 h
*Radiation treatment duration (days, median, range)*	30 (28 – 37)
*Chemotherapy before radiotherapy (patients, %)*	91 (91)
Cycles (n, median, range)	2 (1 – 4)
Carboplatin	76 (76)
Cisplatin	15 (15)
Gemcitabine	40 (40)
Pemetrexed	36 (36)
Navelbine	12 (12)
Etoposide	2 ( 2)
Taxotere	1 ( 1)
*Durvalumab, consolidation post radiotherapy (patients, %)*	
Started	36 (36)
Completed at 12 months, without toxicity	11 (11)
Incomplete, until now well tolerated	12 (12)
Premature termination	13 (13)
PD-L1-negativity	4 (4)
Progression of disease	4(4)
Toxicity	2 (2)
Intercurrent death	2 (2)
Patient request	1 (1)

*PT–Ø = Diameter of the primary tumor (mean of three perpendicular diameters);

**To a restricted nodal volume

Dose-volumetric parameters of organs at risk are listed in Table 3. Noteably, patients were treated with pulmonary V20 (both lungs) up to 53%, and mean lung doses up to 26 Gy. In 20% of all patients the pulmonary QUANTEC-constraints were surpassed.

**Table 3:** Dose-volumetric parameters of organs at risk (median, range); doses specified as physical doses

*Lung*	
V 20* ipsilateral (%)	37 (7–64)
V 20 contralateral (%)	19 (0–52)
V 20 both lungs (%)	28 (4–53)
V 5 both lungs (%)	61 (14–100)
D mean (Gy)	15.0 (3.8–26.0)
*Proximal bronchial tree*	
D mean (Gy)	47.6 (2.8–70.4)
D max 0.1 ccm (Gy)	82.4 (52.9–95.8)
V 75 (%)	7 (0–46)
V 85 (%)	0 (0–32)
*Heart*	
D mean (Gy)	8.6 (0.2–38.5)
V 5 (%)	33 (0–100)
V 30 (%)	6 (0–60)
V 50 (%)	1 (0–28)
*Esophagus*	
D mean (Gy)	22.1 (6.7–43.2)
D max 0.1 ccm (Gy)	63.8 (30.0–90.7)
V 60 (%)	2 (0–36)
*Spinal cord*	
D max (Gy)	41.4 (16.7–49.6)

Vx = Volume of an organ exposed to ≥ x Gy

91 patients (91%) received induction chemotherapy. 1, 2, 3 and 4 cycles were given in 1, 85, 3 and 2 patients, respectively (Table 2). 11 patients finished 1 year maintenance therapy with Durvalumab.

Median follow up time for all patients is 19.4 months (3.4–66.5 m.), for patients alive 27.0 months (3.4–66.5 m.). One patient was lost to follow-up; he was disease-free without late toxicity at his last visit at 26 months, and was censored at this time.

### Acute toxicity

Table 4 shows the non-hematologic toxicities.

**Table 4:** Acute (A) and late (B) non-hematologic toxicity (CTCAE,v 4.3), n = 100; n (%)

		Grade 1	Grade 2	Grade 3	Grade 4	Grade 5
A	Pneumonitis	n.a	12 (12)	2 (2)	–	–
	Esophagitis	12 (12)	57 (57)	3 (3)	–	–
B	Bronchial stricture/Atelectasis	n.a	2 (2)	–	–	–
	Hemorrhage	–	–	–	–	2 (2)

CTCAE = Common Terminology Criteria for Adverse Effects; n.a. = not assessed

Grade 2 and 3 pneumonitis occurred in 12 and 2 patients, respectively; no grade 4 or 5 pneumonitis was observed. Both patients with grade 3 pneumontis presented with a second NSCLC and were treated for a first lung cancer 2–3 years ago; one patient with lobectomy, the other with radiotherapy. Pulmonary bi-, ipsi- and contralateral V20 were below the median figures for the entire cohort. Pneumonitis was medicated with steroids in declining doses for 8 and 10 weeks, respectively.

For grade 2 patients, the median of all pulmonary dosimetric parameters coincided with the median values for all patients.

An increased rate of pneumonitis in patients receiving durvalumab consolidation was not observed; only two out of 36 patients showed grade 2 pneumonitis.

The risk of pneumonitis grade ≥ 2 was 14/100 = 14% in our study and 250/836 = 29.8% in the comparative study by Palma et al. [11], which was found to be statistically significant (Fisher’s Exact Test, two-sided, p < 0.0006).

Esophagitis grade 1, 2 and 3 was observed in 12, 57 and 3 patients, respectively. In all patients symptoms disappeared after a few weeks of analgetic therapy.

### Late toxicity

In two patients with central tumors, grade 2 atelectasis of the middle lobe occurred 6 and 16 months, respectively after therapy. Both patients were treated with 79.2 Gy. V75 of the proximal bronchial tree (PBT) was 39% and 13%, respectively.

2 patients with central tumors in tight vicinity to great vessels suffered lethal hemorrhages. They were treated with 84.6 Gy and 73.8 Gy, respectively. The events occurred 7 and 9 months after the end of therapy. As a treatment correlation cannot be excluded, they were scored grade 5.

### Survival time

57 patients are alive and 43 dead. Overall survival (OS) rate at 2 and 3 years for all patients is 56% and 49%, respectively. For stage III and IVA patients (n = 88) and for those with ‘RTOG-0617 inclusion criteria’ (n = 38), the median OS amounts to 46.6 months and 50.0 months, respectively.

## Discussion

To 96% of all patients, the scheduled radiation doses were able to be applied.

### Pneumonitis

Is the primary end point of the study. Though applying unusual high total doses, no grade 4 or 5 toxicity occurred; and pneumonitis grade 2 and 3 was observed in 12% and 2% of patients only. Grade 3 pneumonitis occurred exclusively in patients treated for a previous lung cancer some years ago.

For comparison with a representative cohort of patients, the largest study evaluating radiation pneumonitis in LA-NSCLC was chosen [11]. 836 patients were analyzed with individual patient data. Radiation was performed in 3D-conformal or IMRT mode (exact fractions not specified). The median total dose was 60 Gy, fractional doses were ≤ 2 Gy in 94% of patients. Patients were treated concurrently with chemotherapy. Grade ≥ 2 pneumonitis resulted in 29.8%, grade 5 in 1.9% of patients. An aposteriori sample size analyses showed that the two-sided Fisher’s Exact test achieved a power of 95.6% to compare the risk of pneumonitis with grade ≥ 2 in our study (14/100 = 14%) with the results of the meta-analysis (250/836 = 29.8%).

The two patient collectives are not ideally identical. Patients in the meta-analysis were treated before 2013, partly with 3-D conformal technique. On the other hand, in our patients total doses were definitely higher. Concurrently applied chemotherapy cannot explain the higher toxicity in the comparative cohort, as shown in another meta-analysis of 1405 patients, revealing no difference in the incidence of pneumonitis between concurrent and sequential chemo-radiotherapies [17]. More recent studies of patients treated predominantly with IMRT show better results. But still the incidence of pneumonitis grade ≥ 3 lies between 2 and 7%, though just 60–74 Gy were applied [18–20]. Modern studies of patients treated with VMAT, applying 60–70 Gy, show an incidence of pneumonitis grade ≥ 2 between 22 and 28% [21–23].

In our opinion the low pneumonitis rate in our patients can be explained by the mode of sparing normal lung tissue, i.e. by maximal ipsilateral sparing. It is achieved by tight margins and by appropriate pulmonary dose distributions as described. Pneumonitis primarily arises in ipsilateral lung, where the gross of dose is applied. Therefore, ipsilateral normal lung should be spared, naturally without spillover of toxicity to the contralateral side.

A critical point is the level of pulmonary dose constraints. We built up from experiences with target splitting [9]. In a series of 150 patients (24], patients with upper lobe-, middle lobe- and central lower lobe tumors (n = 130) were treated with V20 up to 50% and patients with peripheral lower lobe tumors (n = 14, basal lateral excluded) up to 42%; resulting in totally 8% of patients with grade 3 pulmonary toxicity recovering well with steroids. Only patients with basal *lateral* lower lobe tumors experienced grade 4/5 events, namely 3 of 5 patients, with V20 ranging between 30 and 53%. In the here presented VMAT series, patients with upper-, middle—and central lower lobe tumors (n = 78) were treated with V20 (total lung) up to 53% and patients with peripheral lower lobe tumors (n = 22) up to 47%, resulting in the low pneumonitis rate as described. In 20 patients (20%) the upper limit of QUANTEC for V20 (35%), and in 4 patients the upper limit for mean lung dose (23 Gy) was surpassed. QUANTEC-recommendations (V20 ≤ 30–35%, Dmean ≤ 20–23 Gy) are based on 3D-conformal therapies, usually sparing contralateral lung [25]. Constraints are driven by the level of e.g. bilateral V20, causing a symptomatic pneumonitis. In ‘contralateral lung sparing’ approaches pneumonitis could arise at a relatively low target dose, even when V20 (total lung) is kept low. In contrast, ‘ipsilateral lung sparing’ approaches could result in a safe higher target dose and – as it seems—also in a higher bilateral V20 as a constraint relevant value. Thus, *a constraint is a figure dependent on the methodical approach*; an insight also stated in the QUANTEC-report [25].

Apart from the comparison to current normofractionated schedules, toxicity results of our patients compare favorably also to dose escalation studies by hypofractionation. 23.8% of patients in the RTOG 1106 trial showed grade ≥ 3 pulmonary events [26]. In the PET-boost trial, acute and late non-hematological toxicity grade ≥ 3 occurred in 29% and 24%, respectively [27].

### Low dose bath

Applying IMRT or VMAT techniques, potential adverse effects of larger volumes in the pulmonary low dose range are an issue [6].

Many investigations deal with the occurrence of radiation pneumonitis and several reports are on 3D–treatments of LA-NSCLC. Common features therein are target doses of about 60 Gy in conventional fractionation, applied with intended sparing of the contralateral lung and combined with chemotherapy concurrently [1, 3, 28]. Severe, symptomatic pneumonitis in up to 32% of treated patients is a frequent event in these treatments. Wang et al. [3] describe V5 as the most significant factor associated with pneumonitis; the incidence of grade ≥ 3 pneumonitis in patients with V5 < 42% and those with V5 > 42% was 3% and 38%, respectively. The authors state however, that in view of high correlation among different lung volumes, it is not possible to determine the most important dose range for inducing grade ≥ 3 pneumonitis. Schallenkamp et al. report V10 and V13 as best predictors of pneumonitis risk; but again, the most predictive method is not clear, as most dose-volume metrics are functions of each other [28]. In the series reported by Barriger et al. MLD > 18 Gy and maintenance chemotherapy with docetaxel were predictive for pneumonitis, but not V5 through V30 (1].

In two studies, comparing 3D-conformal and IMRT techniques, despite a significantly higher V5 level in the IMRT groups, the incidence of pneumonitis using IMRT is lower [2, 18].

In the meta-analysis by Palma et al., the comparative cohort of this study, factors predictive of pneumonitis were V20 and carboplatin/paclitaxel chemotherapy, not V5 [11]. Likewise, in a secondary analysis of RTOG-0617, V5 was not associated with any kind of ≥ grade 3 toxicity [18]. The authors argue against using lung V5 for IMRT plan optimization, because lowering V5 can potentially lead to less conformity in the intermediate and high dose region, both of which are important objectives confirmed in their study.

In the here presented study, a pulmonary V5 > 60% and > 80% resulted in 51 patients (51%) and 19 patients (19%), respectively; until now, we did not observe any adverse effect associated with this low dose exposure.

### Esophagitis

Grade 1, 2 and 3 occurred in 12%, 57% and 3% of all patients, respectively. The high percentage of esophagitis grade 2 compared to grade 1 is due to the use of the CTC–definition, in which grade 1 esophagitis corresponds to ‘clinical or diagnostic observation only, intervention (i.e. medication) not indicated’. Under this definition, many patients taking some ‘mild’ analgesic for a short period only, had also been scored grade 2. This differentiates to the RTOG definition, where grade 1 corresponds to ‘using anesthetics or non-narcotic analgesics’.

### Proximal bronchial tree (PBT)

Dose-volumetric parameters regarding PBT can be seen in Table 3. In all patients with central tumors, some asymptomatic narrowing of the great bronchi can be observed. However, in only 2 patients bronchial stricture grade 2 with atelectasis of the middle lobe occurred. These events emerged 6 and 16 months after treatment, with moderate symptoms of dyspnea and cough.

The literature regarding toxicity of PBT in fractionated radiotherapy is scarce. Miller et al. describe grade 4/5 toxicity in 6% of stage III patients, treated with 80 Gy in 1.6 Gy bid fractions in 3D-conformal mode [29]. Wang et al. correlated PBT toxicity to dose-volume parameters [30]. 100 patients were treated with 60–85,5 Gy in 3D-conformal technique, with concurrent chemotherapy. 9% grade 2 + toxicity occurred (3% grade 4). V75 was the most significant dosimetric parameter, with a threshold of 12% for prediction of grade 2 + toxicity. In our series PBT V75 > 12% resulted in 38 patients; V85 ≥ 10% and ≥ 20% emerged in 12 and 5 patients, respectively. Only in 2 patients—with V75 13% and 39%, respectively and V85 of 0%—grade 2 toxicity as described, occurred. In our opinion this high PBT tolerability is primarily based on avoidance of intermediate and high dose radiation to PBT-adjacent normal tissues, inherent in our approach.

Apart from toxicity, in our experience pretherapeutic atelectasis in about half of the patients temporarily clear up, but by the majority later re-appear and persist.

### Late toxicity

Besides two patients with grade 2 late toxicity of bronchial stricture/ atelectasis, two patients with central tumors suffered lethal hemorrhages. They died 7 and 9 months after the end of radiotherapy with 84.6 Gy and 73.8 Gy, respectively. In both cases a recurrent tumor was not detected previously. Autopsies were not performed. The most probable cause of these hemorrhages is weakening of a vessel wall by retraction of originally vessel-infiltrating tumors. In this sense, a treatment relationship could not be excluded, therefore the events were scored grade 5. The ‘safe’ application of high doses also to central tumors is underlined by the previous DART-bid series by target-splitting [24]: Only 2 out of 150 patients died of a possibly treatment related hemorrhage. One patient with a peripheral tumor and another with a central tumor, both treated with 79.2 Gy. However, 21 patients with central tumors were treated with doses ≥ 82.8 Gy (median 84.6 Gy, range 82.8–87.3 Gy); and lethal hemorrhages, fistulae, cartilaginous necrosis or similar toxicities did not occur.

### Elective nodal irradiation (ENI)

As in the previous target-splitting series, also in this VMAT-series a restricted ENI was performed as described in the ‘methods’-section. The rationale was: in some ‘involved field series’ (treatments without ENI), isolated elective nodal recurrences are described in up to 9% of patients [31, 32]; and microscopic spread of tumor cells in adjacent lymph nodes in ‘direction of lymph flow’ occurs probably also in PET-staged patients.

In contrast to ‘classical ENI’, PTVs in our patients were restricted, beam arrangements highly conformal and total doses lowered to 45 Gy. The recently published PET-Plan study however describes a lower risk of locoregional progression in patients treated without ENI [33]. This let us think about omitting ENI in the future, especially in patients treated with immunologic agents.

### Sequential versus concurrent chemo-radiotherapy

At present, concurrent chemo-radiotherapy (60 Gy, 2 cycles), is regarded standard of care for LA-NSCLC. However, for reasons of tolerability only about 30% of all patients can be treated with the concurrent approach [34].

Doubtless, 60 Gy combined with chemotherapy concurrently give better results than combined sequentially; but this does not mean, that higher radiation doses combined with chemotherapy sequentially cannot overrule 60 Gy combined concurrently. Recently, a meta-analysis of randomized trials, comparing radiation regimens with different time-corrected total doses was published [35]. Higher total radiation doses in concurrent approaches resulted in poorer survival. Where radiation was given without chemotherapy or in a sequential mode, progressively higher radiation doses resulted in progressively longer survival. Moreover, no upper dose level was found, above which there was no further benefit.

Survival, as locoregional tumor control, is beyond the scope of this article. However, the survival results of our patients compare strongly favorably to the outcome of concurrent therapies: Median OS for all stage III patients, and for those with ‘RTOG 0617 inclusion criteria’ is 46.6 and 50.0 months, respectively. For comparison, median OS in the favorable 60 Gy arm of RTOG 0617 amounts to 28.7 months [36]; and in the Pacific trial – with a twice selected patient cohort—to 47.5 months [37].

### Limitations

Creating the trial concept, we were conscious of the advantages of a randomized trial. However, performing such a trial appeared unrealistic. Thus we decided on a comparison with data from the literature.

As this trial investigated primarily radiotherapeutic objectives, the protocol regarding chemotherapy was not stringent. In particular, choice of chemotherapeutic agents was left to the discretion of the various referring departments.

## Conclusions

In comparison with results from the literature, maximal sparing of ipsilateral normal lung tissue in high dose accelerated radiotherapy and sequential chemotherapy, lowers the incidence of pneumonitis significantly. Also rates of further toxicities are low.

## Data Availability

The datasets used and/or analysed during the current study are available from the corresponding author on reasonable request.

## Abbreviations

NSCLC: Non-small cell lung cancer: DART-bid (dose-differentiated accelerated radiation therapy, 1.8 Gy twice daily)
VMAT: Volumetric arc therapy
LA-NSCLC: Locally advanced non-small cell lung cancer
Gy: Gray
ITV: Internal target volume
PTV: Planning target volume
CTV: Clinical target volume
GTV: Gross tumor volume
ICRU: International commission on radiation units
UICC/AJCC: Union for international cancer control/American Joint Commission on Cancer
QUANTEC: Quantitative analysis of normal tissue effects in the clinic
RTOG: Radiation therapy oncology group
PD-L1: Programmed death ligand 1
FTG-PET: 18-Fluorodeoxyglucose-positron emission tomography
CT: Computed tomography
MRI: Magnetic resonance imaging
4 D: 4–Dimensional
MV: Megavolt
CTCAE: Common terminology criteria for adverse effects
PBT: Proximal bronchial tree
OS: Overall survival
IMRT: Intensity modulated radiotherapy
MLD: Mean lung dose
n.a.: Not assessed
Vx: Volume of an organ exposed to ≥ x Gy
COPD: Chronic obstructive pulmonary disease
NSC—n.o.s.: Non-small cell—not otherwise specified
PT–Ø: Diameter of the primary tumor
